# Draft Genome Sequences of Two *Janthinobacterium* *lividum* Strains, Isolated from Pristine Groundwater Collected from the Oak Ridge Field Research Center

**DOI:** 10.1128/genomeA.00582-17

**Published:** 2017-06-29

**Authors:** Xiaoqin Wu, Adam M. Deutschbauer, Alexey E. Kazakov, Kelly M. Wetmore, Bryson A. Cwick, Robert M. Walker, Pavel S. Novichkov, Adam P. Arkin, Romy Chakraborty

**Affiliations:** a Earth and Environmental Sciences, Lawrence Berkeley National Laboratory, Berkeley, California, USA; b Environmental Genomics and Systems Biology Division, Lawrence Berkeley National Laboratory, Berkeley, California, USA; c Program in Comparative Biochemistry, University of California, Berkeley, California, USA; d Department of Bioengineering, University of California, Berkeley, California, USA

## Abstract

We present here the draft genome sequences of two* Janthinobacterium lividum* strains, GW456P and GW458P, isolated from groundwater samples collected from a background site at the Oak Ridge Field Research Center. Production of a purple pigment by these two strains was observed when grown on diluted (1/10) LB agar plates.

## GENOME ANNOUNCEMENT

The genus *Janthinobacterium* includes rod-shaped, Gram-negative, motile, aerobic bacteria that are known to produce the water-insoluble purple pigment violacein (1, 2). Violacein has been known to have antimicrobial and antiviral properties (1, 3, 4), inhibiting the growth of bacteria (5, 6) and providing bacteria protection against predators (7, 8). To date, only a few environmental isolates of *Janthinobacterium* spp. have been sequenced (2), and these were isolated from lake sediments (9) and soils (10, 11), mostly in cold habitats (12–16). Until now, no sequenced representatives of *Janthinobacterium* spp. have been available from the subsurface aquatic environment. In this study, two violet-pigmented *J. lividum* strains were isolated on diluted (1/10) LB agar plates, from groundwater samples collected from two wells (GW456 and GW458) located at the background area of the Oak Ridge Field Research Center, Oak Ridge, Tennessee, USA.

Genomic DNA was extracted using the PureLink Genomic DNA mini kit (Invitrogen). Genomic sequencing libraries of the two *J. lividum* strains, GW456P and GW458P, were prepared using the NEBNext DNA library prep kit for Illumina (New England Biolabs). Briefly, 1 µg of genomic DNA was fragmented by ultrasonication to an average size of 800 bp with a Covaris S220 focused ultrasonicator. After end-repair, A-tailing, and ligation of the adapter, we size-selected 800-bp products with AMPure XP beads. The sequencing libraries were quantified on a bioanalyzer with a DNA1000 chip (Agilent). We performed paired-end sequencing (2 × 150 bp) on an Illumina MiSeq using a MiSeq version 2 reagent kit (300 cycles).

The raw reads were assembled using the A5 microbial assembly pipeline version 0.0.4 (17), implemented within the U.S. Department of Energy’s Systems Knowledge Database (Kbase; http://www.kbase.us) (18). This pipeline automates the processes of read cleaning, error correction, contig assembly, crude scaffolding, misassembly correction, and final scaffolding with stringent parameters repairing previously broken contigs. The draft genome sequence of strain GW456P was 6.27 Mb in length, in 78 contigs, and the total G+C content was 62.89%. For strain GW458P, the genome size was 6.29 Mb, consisting of 156 contigs with a total G+C content of 63.29%.

Automated annotation for each genome was performed using the Joint Genome Institute’s Integrated Microbial Genomics (IMG) version 4.12.0 annotation pipeline (19). Proteins related to violacein biosynthesis were identified in both strains by comparison with the VioA, VioB, VioC, VioD, and VioE proteins from *Janthinobacterium* sp. HH01 (2) using the GenomeExplorer program (20). We also identified three genes known to regulate violacein biosynthesis (2): the sensor kinase *jqsS*, the response regulator *jqsR*, and the autoinducer synthase *jqsA*. Upstream regions of the *vioABCDE* operons in both strains contain the inverted repeat TTGATATTTATCAA, which coincides with the published JqsR binding motif (21). Thus, we propose that the expression of violacein biosynthesis operons in strains GW456P and GW458P is dependent on quorum sensing. These genomes are useful for exploring the physiology and regulation of violacein pigment production by these two *J. lividum* strains.

### Accession number(s).

Genomes of *J. lividum* strain GW456P and *J. lividum* strain GW458P have been deposited at DDBJ/ENA/GenBank under the accession numbers NEHB00000000 and NEGZ00000000, respectively.
